# The development and function of the brain barriers – an overlooked consideration for chemical toxicity

**DOI:** 10.3389/ftox.2022.1000212

**Published:** 2022-10-18

**Authors:** Kiersten S. Bell, Katherine L. O’Shaughnessy

**Affiliations:** ^1^ US Environmental Protection Agency, Public Health Integrated Toxicology Division, Center for Public Health and Environmental Assessment, Research Triangle Park, NC, United States; ^2^ Oak Ridge Institute for Science Education, Oak Ridge, TN, United States

**Keywords:** brain development, blood-brain barrier, blood-cerebrospinal fluid barrier, environmental contaminants, mechanisms of neurotoxicity, perfluoroalkyl substances (PFAS), neurodevelopmental disorders, bisphenol

## Abstract

It is well known that the adult brain is protected from some infections and toxic molecules by the blood-brain and the blood-cerebrospinal fluid barriers. Contrary to the immense data collected in other fields, it is deeply entrenched in environmental toxicology that xenobiotics easily permeate the developing brain because these barriers are either absent or non-functional in the fetus and newborn. Here we review the cellular and physiological makeup of the brain barrier systems in multiple species, and discuss decades of experiments that show they possess functionality during embryogenesis. We next present case studies of two chemical classes, perfluoroalkyl substances (PFAS) and bisphenols, and discuss their potential to bypass the brain barriers. While there is evidence to suggest these pollutants may enter the developing and/or adult brain parenchyma, many studies suffer from confounding technical variables which complicates data interpretation. In the future, a more formal consideration of brain barrier biology could not only improve understanding of chemical toxicokinetics but could assist in prioritizing environmental xenobiotics for their neurotoxicity risk.

## Introduction

Exposure to environmental contaminants during development may have persistent negative impacts on human health across the lifespan (Grandjean and Landrigan, 2006; Landrigan and Goldman, 2011; Grandjean and Landrigan, 2014). Recently, the link between early life exposures to pollutants and neurodevelopmental disorders (NDDs) has received more public attention (Diamanti-Kandarakis et al., 2009; Gore et al., 2014). The National Health Interview Survey, a survey of American civilian noninstitutionalized households, found approximately one in six children aged 3–17 were diagnosed with one of ten specified developmental disabilities (e.g., autism, attention-deficit/hyperactivity disorder, and learning disabilities) between 2009 and 2017 (Zablotsky et al., 2019). Between 2009–2011 and 2015–2017, there were also significant increases in the prevalence of all three NDDs in American children (Zablotsky et al., 2019). While these observed increases could be attributed to increased awareness in health care providers and parents, growing evidence suggests that exposure to environmental contaminants, in addition to genetic and psychosocial factors, may play an overlooked role in altering brain development (Grandjean and Landrigan, 2006; Axelrad et al., 2013; Grandjean and Landrigan, 2014; Landrigan et al., 2019).

Human neurodevelopment is an intricately choreographed process that begins 3 weeks post-conception and concludes in early adulthood (Stiles and Jernigan, 2010; Budday et al., 2015). This protracted maturation period poses unique challenges when attempting to understand NDDs, as there are complex and time-dependent sequences of chemical messages required for normal development (Diamanti-Kandarakis et al., 2009). For example, a NDD may be diagnosed long after perturbation of the causative developmental pathway (Diamanti-Kandarakis et al., 2009). Understanding these windows of susceptibility is especially challenging when trying to understand how environmental contaminants may influence the developing brain. Beginning as early as conception, a person could be exposed to a multitude of industrial chemicals that are used in products like children’s toys, food preparation and packaging, personal care products, and household agents (O'Shaughnessy et al., 2021). While having economic and consumer benefit, these chemicals are used ubiquitously in communities and associated with air pollution, electronic waste, flame retardants, plastics, and pesticides (Diamanti-Kandarakis et al., 2009; Gore et al., 2014; O'Shaughnessy et al., 2021). Although there are at least tens of thousands of manufactured compounds currently in the chemical universe, very few have undergone toxicological evaluation (Judson et al., 2009; Rayasam et al., 2022) and even fewer have undergone developmental neurotoxicity testing. Grandjean and Landrigan (2006) published a systematic review identifying over 100 industrial chemicals known to be neurotoxic to humans, five of which could be classified as developmental neurotoxicants (arsenic, lead, methylmercury, polychlorinated biphenyls, and toluene). Their 2014 report found six more developmental neurotoxicants (chlorpyrifos, dichlorodiphenyltrichloroethane, fluoride, manganese, polybrominated diphenyl ethers, and tetrachloroethylene) (Grandjean and Landrigan, 2014). Several other reviews and meta-analyses have found associations between industrialized chemicals and developmental neurotoxicity (Heyer and Meredith, 2017; Landrigan et al., 2019; Iqubal et al., 2020) both in population-based studies and animal models.

Within the field of developmental neurotoxicology, a feature of the central nervous system that is often overlooked is the development and function of the brain barriers. These interfaces separate the brain from other bodily fluids (e.g., blood, cerebrospinal fluid), maintain homeostasis, and protect against toxic molecules and infection. However, there is a large data gap in determining whether/when industrial chemicals can cross into the brain, due in part to a common misconception that the developing brain has an immature and therefore “leaky” (i.e., permeable) barrier system. Many recent reports mention that neurotoxicants are easily transported into the young brain with little, if any, discussion as to what processes purportedly take place (see Ek et al., 2012 for review). Surprisingly, the mechanism of toxicity for even the best studied neurotoxicants is still not clear (Ek et al., 2012), which ultimately begets the question: how are environmental contaminants entering the young brain, if at all? Moreover, could exposure to some environmental contaminants alter brain barrier function? Alterations in blood- and cerebrospinal fluid-brain barrier integrity is now recognized as an important component of epilepsy (Baruah et al., 2019), and its dysfunction could contribute to disorders like autism (Kealy et al., 2020), attention-deficit/hyperactivity disorder (Medin et al., 2019), and schizophrenia (Najjar et al., 2017; Greene et al., 2018; Pollak et al., 2018; Kealy et al., 2020). Progress in brain barrier research has also shown that the adult barrier system is a target of environmental toxicants (Zheng et al., 2003; Zheng and Ghersi-Egea, 2020). Thus, it is possible that early-life exposure to some chemicals could lead to pathophysiological changes in brain barrier function, which could result in NDDs or other brain disorders. However, there is little work that investigates this hypothesis.

In this review, we briefly outline the rich, 200-year history of brain barrier biology. We next discuss the cellular and physiological properties that underlie the function of the brain barrier systems, and present evidence to show that the developing brain possesses functional blood and cerebrospinal fluid barriers. Finally, we critically review the neurotoxicological literature that may provide evidence of environmental contaminants crossing into brain tissue. While it is widely believed in environmental toxicology that the developing brain is more susceptible to neurotoxic insults than the adult due in part to inadequate brain barriers, there is a lack of evidence to support this assumption. Alternatively, there are convincing data that the barriers form and possess functionality during embryogenesis in both humans and experimental models. We challenge readers in the field to reevaluate their understanding of these fluid interfaces, as in many contexts the brain barriers will determine the neurotoxic potential of a chemical exposure.

## History of the brain barriers

The physiological compartmentalization of the brain and spinal cord has been described for centuries and is widely accepted across scientific disciplines. Dating back to the late 1600s, the London physician Humphrey Ridley is likely one of the first to recognize and describe the brain’s ability to obstruct certain compounds from crossing in the adult brain (Ridley, 1695). Almost 200 years later, the brain’s ability to selectively filter substances was more formally established during an attempt to cure African sleeping sickness caused by the protozoa, trypanosomes (Bentivoglio and Kristensson, 2014). Trypanosomes readily cross into the central nervous system (Myburgh et al., 2013), eliciting agonizing neurological symptoms if left untreated. In the late 1800s, the medical researcher Paul Ehrlich attempted to kill the parasite with dyed antiprotozoal agents (trypanocides) to treat infection (Bentivoglio and Kristensson, 2014). Instead, he accidentally discovered that the trypanocides could *not* traverse into the brain tissue. Through subsequent studies in which both Ehrlich and his student Edwin E. Goldman injected dyes in rodents, it was revealed that the whole body was stained except for the brain and spinal cord (Ehrlich, 1885; Goldmann, 1909). Ehrlich initially believed this was due to the brain tissue lacking affinity for the dyes as opposed to a formal barrier, but this work nonetheless marked a seminal moment in neuroscience. To further their findings, Goldman conducted an additional study in which he injected trypan blue, another dye, directly into the brain and found that it was maintained within the neural tissue, suggesting that some type of boundary existed between the blood and brain (Goldmann, 1913). However, “barrier” was not used to describe the brain’s selectivity phenomenon until 1918 by Stern and Gautier (Stern and Gautier, 1918), although many publications wrongfully ascribe the term to Lewandowsky’s paper where he recounted the relative impermeability of the brain’s vasculature (Lewandowsky, 1900). These initial studies were pivotal in the recognition of the blood-brain barrier in the adult, and the traditional method of injecting dyes either intravenously or intraperitoneally in animal models to determine the functionality of the brain barriers is still in practice today. It has since been established that the adult brain is well-protected by several brain barriers through elegant ultrastructural, immunohistochemical, and functional studies.

Unlike the adult, the notion of fully functioning barrier system in the fetus, newborn, and child continues to be highly disputed amongst physicians, researchers, and governing agencies despite the mounting evidence collected over the last 100 years (Neuwelt et al., 2011; Ek et al., 2012; Saunders et al., 2018, 2019). Weed (1917) conducted one of the first developmental studies and demonstrated that the central nervous system is a closed compartment as early as gestational day nine in pig embryos (full term ∼115 days), as evidenced by restriction of his injected tracer (sodium ferrocyanide) to the cerebrospinal fluid (CSF). Wislocki (1920) injected trypan blue into the amniotic sacks of guinea pig embryos from mid-gestation to full term and noted that the dye stained tissues throughout the body except for the brain. Many dye studies repeatedly illustrated that dyes do not enter the developmentally immature brain as long as an appropriate amount of dye was used (e.g., Stern and Rapoport, 1928; Cohen and Davies, 1938; Gröntoft, 1954; Grazer and Clemente, 1957; Millen and Hess, 1958; Moos and Møllgård, 1993). Interestingly, one study was conducted in human fetuses as young as 10 weeks post conception (∼5 cm in length), and again the whole brain was not stained unless a state of hypoxia had occurred prior to collection (Gröntoft, 1954). The first papers to ostensibly provide support for a developmentally dysfunctional blood-brain barrier were conducted by Gerhard Behnsen, in which he injected excessive amounts of dye in mice (Behnsen, 1926, 1927). Penta (1932) also used large volumes of injected dye in newborn rabbits. Although the dye stained brain tissue which suggested the brain barriers were not functioning, most of the animals died from toxic effects of the dye. In response to Behnsen and others, Stern et al. (1929) underscored the concern of injecting too much dye in young animals, as superseding a dye’s binding capacity results in physiological instability, overt neurotoxicity, and eventual entry in the brain (see Saunders et al., 2015 for further review). So, although some studies report the functional immaturity of the brain barriers, these observations were later attributed to experimental artifact. Once technical parameters were adjusted, functionality could again be demonstrated during development across multiple species.

Despite these accepted biological principles, toxicology publications within the last 10 years continue to imply that the barrier systems during development are permeable due to their immaturity (see Grandjean and Landrigan 2014; Heyer and Meredith 2017; Rock and Patisaul 2018; Iqubal et al., 2020), and thus xenobiotics are assumed to enter the brain tissue without any, or potentially mis-cited, supporting evidence (see Ek et al., 2012 for review).

## What constitutes the brain barriers?

The brain is the most complex organ in the body that regulates all physiological processes at some level. As such, it is ensconced by several protective barriers. While there are at least six barriers (Saunders et al., 2018), the focus of this review will be on the two barriers of most medical importance: the blood-brain barrier (BBB) and the blood-cerebrospinal fluid barrier (BCSFB). We will also be briefly discussing the transient fetal cerebrospinal fluid-brain barrier, which may have an important role during development.

### The blood-brain barrier

Once absorbed in the bloodstream, a substance (e.g., xenobiotics, gases, nutrients) circulates through the vascular system. The vascular system is incredibly heterogenous in structure and function around the body and within organs (Aird, 2007a; b), with the capillaries and postcapillary venules of the central nervous system (CNS) being particularly unique (Daneman and Prat, 2015). The specialized blood vessels of the CNS are composed of tightly packed endothelial cells and, unlike most other vessels around the body, form a continuous non-fenestrated boundary. The lack of capillary pores prohibits diffusion of most substances. In turn, this isolates the circulating blood from the CNS, including the brain. Mammalian brains are highly vascularized and contain over 100 million vessel segments (Kirst et al., 2020); however, it is the cerebral capillaries (<10 μm diameter) (Tong et al., 2020) that constitute the BBB and corresponds to a surface area of 15–25 m^2^ in adult humans (Lauwers et al., 2008; Wong et al., 2013). The BBB is highly involved in the transfer of nutrients and drugs to the brain (Abbott et al., 2008; Wong et al., 2013) and acts as both a physical and metabolic barrier (Abbott, 2005; McCaffrey and Davis, 2012). Its series of cellular properties (discussed below) allow it to regulate brain homeostasis and serve as a gatekeeper between the circulating blood, brain interstitium, and parenchyma, by tightly regulating the exchange of blood constituents such as ions, glucose, hormones, and neurotransmitters (Stonestreet et al., 2006; Zlokovic, 2008; Daneman and Prat, 2015).

#### Cellular properties

**FIGURE 1:**
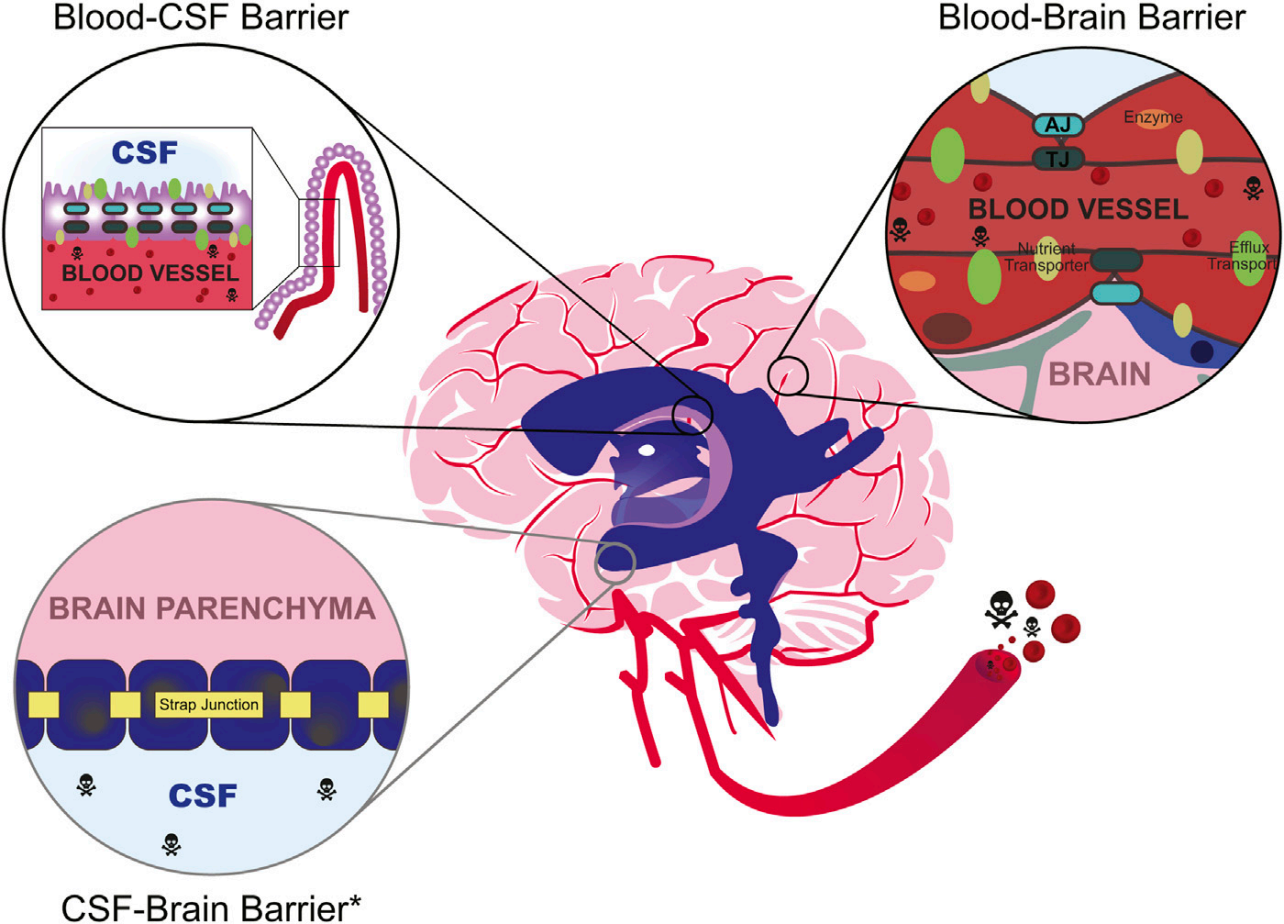
Environmental toxicants (skull and crossbones) commonly enter the bloodstream after inhalation, ingestion, or dermal exposure. While in circulation, they may eventually enter the cerebrovascular system, which is partly composed of the brain barrier systems such as the blood-cerebrospinal fluid (CSF) barrier (top left) and the blood-brain barrier (right). The anatomical location of the blood-CSF barrier occurs within the ventricles (purple) at the choroid plexus (lilac), while the blood-brain barrier occurs at the cerebral capillaries (red). The CSF-brain barrier occurs at the level of the cells lining the brain’s ventricles. Depending on the developmental stage, these cells may be progenitors of the ventricular epithelium (developmental) or primarily ependymal cells (mature). All depicted barriers mechanically obstruct the passage of compounds through either tight junctions (“TJ”, dark green hashes) at the choroid plexus epithelial cells and within endothelial cells, or strap junctions (yellow) at the ventricular epithelial cells. Strap junctions are functionally similar to tight junctions, but they are only present during development (denoted by light gray circle, asterisk, *). As the animal ages, they are replaced by adherens junctions (“AJ”, teal). At the blood barriers, other adhesion molecules (e.g., JAM-A/B/C) and cell junction types (e.g., adherens, teal; gap, not pictured) also help maintain attachment between cells. Select compounds may enter the brain through specialized receptor-mediated transporters (e.g., GLUT1, LAT1; olive ovals). Otherwise, unwanted substrates may be metabolized through metabolic enzymes (orange ovals) and/or discharged back into the bloodstream through efflux transporter proteins (e.g., p-glycoprotein, Pgp; multidrug resistance proteins, MRP; green ovals). The blood-brain barrier is also known to dynamically operate as a system called the neurovascular unit, which includes the coordinated function of additional cellular components like pericytes (blue) and astrocytes (gray). It is important to note that the specific composition of the cell structures varies between the blood-CSF barrier, the blood-brain barrier, and the CSF-brain barrier.

The BBB is one of the biggest challenges in the pharmaceutical industry for its ability to prohibit 98% of drugs from crossing into the brain tissue (Pardridge, 2007), due in part to its cellular properties (Abbott et al., 2006; Abbott et al., 2010). The brain’s capillaries are lined with endothelial cells arranged as a modified simple squamous epithelium and are connected by three cell-cell junctions: tight junctions (i.e., occluding junctions), adherens junctions (i.e., zonula adherens), and gap junctions (i.e., septate junctions). Despite having three different cell junction types, the intercellular tight junctions form the seal between endothelial cells. These seals are created at the apical side (closest to blood) where tight junction transmembrane proteins laterally “stitch” two adjacent cells. This closes the intercellular space, averting paracellular diffusion of molecules from the blood into the brain parenchyma (see Tsukita et al., 2001 for review). The cellular barrier is one of the first lines of defense and manually blocks large molecules from entering the tissue (Figure 1).

**FIGURE 2:**
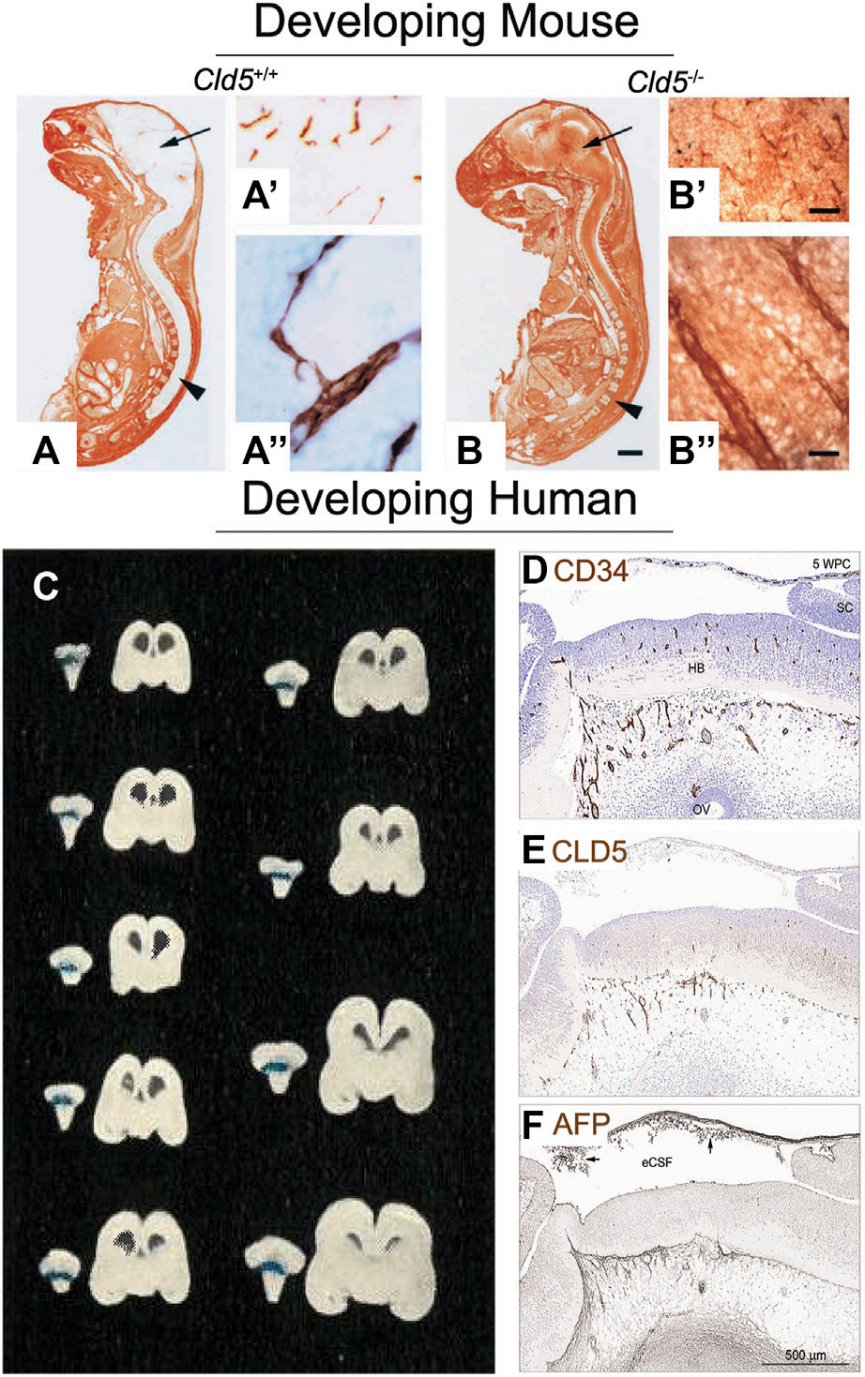
Tight junctions of the blood-brain barrier are present and functional as soon as the brain is vascularized. **(A)** Sagittal frozen section of a gestational day (GD) 18.5 wildtype mouse fetus (*Cld5* ^+/+^) demonstrate functionality of the blood-brain barrier. Primary amine-reactive biotinylated reagent (443 Da, visualized by brown staining) was excluded from the brain parenchyma (arrow) and the spinal cord (arrowhead). **(A′)** Biotinylated reagent (brown) is only visible within the brain’s blood vessels, as visualized at low and **(A′′)** high magnification. This demonstrates that this 443 Da molecule does not cross the BBB, and hence shows its activity **(B)** However, in the *Cld5* knockout mouse (*Cld5* ^−/−^), tight junctions are impaired and biotinylated reagent is visible as brown staining throughout the brain and spinal cord. Extraversion of biotinylated reagent into the brain parenchyma is detectable at **(B′)** low and **(B′′)** high magnification. This shows that even during fetal life, tight junctions are present in the brain and actively occlude substances. *Scale bars*: **(A**,**B)** 2 mm; **(A′)** and **(B′)** 40 μm; **(A′′)** and **(B′′)** 10 μm. Reproduced from Nitta et al., 2003, which was originally published in *Journal of Cell Biology* under a Creative Commons Attribution 4.0 International License (CC-BY) (Nitta et al., 2003). **(C)** Coronal sections of nine human fetuses aged approximately 16 weeks post conception (WPC; 14 cm) to 29 WPC (30 cm) show that trypan blue dye does not permeate the brain tissue after cardiac perfusion, demonstrating an active BBB. Note that blue staining is visible at the choroid plexus of the hind brain, but not in the brain parenchyma. Reproduced from Gröntoft, 1954, which was originally published in *Acta pathologica et microbiologica Scandinavica.* Permissions were obtained from Wiley (Gröntoft, 1954). **(D)** At 5 WPC, consecutive sagittal sections of the human embryonic brain show overlapping staining for blood vessels (CD34; brown staining) and **(E)** tight junctions (CLD5, brown staining). This shows that tight junctions are likely colocalizing with the brain’s vasculature to form the BBB. **(F)** Functionality of the barrier is observed by the lack of endogenous alpha fetoprotein (AFP, 70 kDa) in the forebrain tissue (brown staining). Abbreviations: FB, forebrain; HB, hindbrain; SC, spinal cord, *Scale bar*, 100 μm. Reproduced from © Møllgård et al., 2017, which was originally published in *Scientific Reports* under a Creative Commons Attribution 4.0 International License (CC-BY) (Møllgård et al., 2017).

Tight junction proteins include claudins, occludin, zonula occludens, and junctional adhesion molecules. The claudin family of proteins is recognized as the essential component of paracellular barriers due to experimentation with knockout mice (Saitou et al., 2000) and physiological studies (Furuse et al., 1998) (see Tsukita et al., 2019 for review). The claudin family has 27 identified members (Gunzel and Yu, 2013), 17 of which are present in the human BBB (Berndt et al., 2019). Claudin-5 is the best-studied and appears to be the only tight junction protein whose importance is agreed upon unanimously (Morita et al., 1999; Ohtsuki et al., 2007; Greene et al., 2018). Experimentation with claudin-5 knockout mice (*Cld5* ^−/−^) revealed that homozygous null mutants had morphologically intact tight junctions yet died within hours of being born. The BBB of the affected mice lost its ability to obstruct small molecules from passing into the tissue, as demonstrated by primary amine-reactive biotinylated reagent (443 Da) crossing into the brain parenchyma (Figures 2A–Bʹʹ) (Nitta et al., 2003). In addition, a viral knockdown mouse model against claudin-5 demonstrated that a reduction in claudin-5 induced BBB disruption as characterized by increased amounts of biotin (600 Da) and fibrinogen (340 kDa) in brain tissue; these mice also experienced seizures and behavioral changes (Greene et al., 2018). Other important proteins help constitute the BBB including connexins and endothelial immunoglobulin-like cell adhesion molecules (Zhao et al., 2018). Crosstalk between these aforementioned molecules, amongst others, helps maintain the integrity of the BBB.

The gatekeeping properties of the BBB extend beyond its cell junctions. The BBB dynamically operates as a system: endothelial cells are surrounded by a basement membrane, pericytes, smooth muscle cells, and astrocyte end-feet, forming what is recognized as the neurovascular unit (NVU) (Neuwelt et al., 2011) (Figure 1, also see Saili et al., 2017). The basement membrane is a 20–200 nm (Timpl, 1989) non-cellular matrix between the endothelial cells and astrocytic endfeet, and provides stability to the BBB and blocks blood constituents from traversing (Thomsen et al., 2017). Embedded within the basement membrane are pericytes. Pericytes are required for BBB formation in the fetus (Daneman et al., 2010) and are thought to regulate astrocytes, the basement membrane, blood vessel stability, and blood flow (Armulik et al., 2010; Winkler et al., 2011). Astrocytes are also thought to significantly contribute to, but not induce the formation of the NVU (Abbott et al., 2006; Saunders et al., 2014). In the adult brain, astrocytic endfeet encircle cerebral capillaries, providing an additional barrier for xenobiotics. The astrocytic end-foot is approximately 20 nm away from the capillaries (Paulson and Newman, 1987) and invested in more than 98% of their surfaces (Caley and Maxwell, 1970; Johanson, 1980). Additionally, circulating blood cells such as polymorphonuclear cells, lymphocytes, and monocytes along the vascular lumen are considered components of the NVU (Neuwelt et al., 2011). In all, the interrelationships between the components of the NVU are incredibly intimate, and the regulation of the molecular and cellular events is complex and tightly regulated.

#### Flux-flow dynamics

Blood constituents were previously thought to pass through cerebrospinal fluid first before entering the brain (Pardridge, 2016), but it is now understood that compounds can flow directly from blood vessels into the brain parenchyma. To transfer from blood to cerebral tissue, compounds need to pass through both the luminal (facing blood) and the abluminal (facing brain interstitial fluid) membranes of endothelial cells. Tight junctions deter paracellular movement (see “Cellular properties”), thus *intra*cellular systems along the barrier are important drivers of compound disposition in the CNS. Transporters embedded within endothelial cells control the influx of nutrients and the efflux of wastes, toxicants, and drugs. It is estimated that 10–15% of all proteins in the NVU are transporters (Enerson and Drewes, 2006), and the list of identified transporters at the BBB continues to grow (Tachikawa et al., 2014). Two main categories of transporters have been established: efflux transporters (i.e., ATP-driven membrane transporters) and influx transporters (i.e., receptor-mediated transporters). Efflux transporters from the ATP-binding cassette (ABC) superfamily, including P-glycoprotein (Pgp; *ABCB1, mdr1*), multidrug resistance proteins (MRP; *ABCC* family) and breast cancer resistance protein (BCRP; *ABCG2*), are generally localized to the luminal surface (International Transporter et al., 2010; Morris et al., 2017) and mediate nonspecific transport of hydrophobic compounds. Pgp, MRP1, and BCRP have been the best studied ABC-transporters as they preclude pharmaceutical drug access to brain tissue. Alterations in expression of efflux transporters appear clinically important as diseases associated with inflammation and oxidative stress (e.g., bacterial meningoencephalitis, Parkinson’s, Alzheimer’s, *etc*.) interfere with these transporters (see Roberts and Goralski 2008 for review) (Loscher and Potschka, 2005; Neuwelt et al., 2011). Mechanisms of efflux transport is a highly active area of research to aid in the development of CNS therapeutics. Receptor-mediated transporters also facilitate the transport of specific nutrients across the BBB and have been another target for new drug therapeutics (Trojan horse paradigm) (Pardridge, 2006). Unlike efflux pumps, receptor-mediated transporters are highly specific. Many belong to the solute carrier (SLC) superfamily which is composed of ion channels, exchangers, and passive transporter families.

The speed of blood flow within the blood vessels is also critical for transport. Cerebral blood flow, in concert with the permeability of the compound, directly relate to the rate of CNS penetration (Reichel, 2015). For example, gases such as carbon dioxide, oxygen, and volatile anesthetics diffuse rapidly into the brain and are limited primarily by the cerebral blood flow rate. Cerebral blood flow can be altered through vasodilation and vasoconstriction (Takano et al., 2006; Iadecola and Nedergaard, 2007) and varies in a spatiotemporal and psychosomatic manner around brain (Fenstermacher et al., 1991). However, cerebral blood flow can be largely variable, illustrating the difficulty to define precise reference values. Irrespective of the documented variabilities, blood flow is required to necessitate the transport of substrates to and from the brain.

### The blood-cerebrospinal fluid barrier

With a surface area of 0.02 m^2^ in humans (Dohrmann and Bucy, 1970), the blood-cerebrospinal fluid barrier (BCSFB) is the next largest brain gatekeeper and also plays a role in xenobiotic transport (Lin, 2008; Strazielle and Ghersi-Egea, 2016). Like the BBB, the BCSFB exhibits low paracellular permeability and expresses multiple transporters that aid in the flux-flow dynamics. It is anatomically located around the brain’s cavities at the epithelial layer of the choroid plexus. The choroid plexus is within each of the brain ventricles: the lateral, third, and fourth ventricles. The choroid plexus epithelial cells are responsible for producing most of the cerebrospinal fluid (CSF), the colorless body fluid covering the brain and spinal cord. Adult human CSF volume is estimated to be approximately 150 ml, with choroid plexus epithelial cells secreting around 240–450 ml per day (Sakka et al., 2011). This allows the CSF to turn over three to four times daily, which has important physiological implications such as acting as a waste removal system and a convoy for a variety of compounds to the brain tissue.

#### Cellular properties

The BCSFB is composed of a single layer of specialized cuboidal epithelial cells nested between blood vessels and the CSF, separating the dense network of vasculature from the ventricular system. These epithelial cells contain cilia and microvilli, which force the CSF to circulate from the brain ventricles to the brain stem. Beneath the layer of choroid plexus epithelial cells is an inner core of stromal cells surrounding relatively large capillary blood vessels. Like the BBB, the morphological basis of the BCSFB is at the level of tight junctions. However, the BCSFB tight junctions bind epithelial cells rather than the blood vessel endothelial cells (Figure 1). Unlike the endothelial cells of the BBB, the endothelium within the BCSFB is relatively thinner and fenestrated, allowing for regulated transfer of materials from blood to the interstitial fluid (see “Flux-flow dynamics”). Tight junction proteins in the BCSFB also include the claudins and occludin like the BBB, but the composition is distinct. The role of each tight junction protein has yet to be appropriately defined, but it is believed that claudin-1 and claudin-3 are the prominent transmembrane proteins (Wolburg et al., 2001; Wolburg et al., 2009), and other members of the claudin family are differentially expressed compared to BBB vasculature (Redzic, 2011). Claudin-2 appears specific to the BCSFB and aids in paracellular water movement, and thus is critical to secretion of the CSF (see Bauer et al., 2014 for further review).

#### Flux-flow dynamics

The choroid plexus is also highly vascularized. However, the relatively relaxed cellular properties of its endothelial cells transform the flux-flow dynamics of the choroid plexus from rigid to “leaky” as compared to the BBB (Solár et al., 2020). The composition of the CSF is nonetheless controlled, but the decreased resistance across this cellular barrier allows for plasma solutes to traverse more easily (Redzic, 2011). The choroid plexus produces CSF at a rate of ∼0.29 μl/min/g in human adults (about 1.03–3.00 μl/min/g brain in rodent) (Oshio et al., 2005; de Lange, 2013). This high production rate results in a fast turnover half-life (40–100 min in rat versus 170 min in humans) (Friden et al., 2009), which allows the brain to quickly clear and deliver compounds to brain regions that are in contact with the CSF (e.g., ventricular epithelium) (Johanson et al., 2008). CSF turnover is highly important in the pharmaceutical industry as it affects the drug concentration in the CSF, as well as drug diffusion across various brain compartments (de Lange, 2013). For example, slower flowing CSF results in reduced clearance of substances from the brain and can result in accumulation of potentially toxic molecules; this phenomenon is known as the “sink effect” (Johanson et al., 2008). Because the CSF flow rate is much slower in younger animals, the sink effect was once thought to indicate greater permeability in the developing brain (Saunders et al., 2014).

### The transient CSF-brain barrier

The CSF-brain barrier is not as well studied compared to the blood barriers, but it appears to have at least some implications for normal brain development and its dysfunction is associated with NDDs (Stolp et al., 2011; Stolp et al., 2013). Several studies have also suggested that the presence of the CSF-brain barrier provides increased protection in the fetal brain as compared to the adult (Cavanagh and Warren, 1985; Fossan et al., 1985; Møllgård et al., 1987; Whish et al., 2015).

Junctions between the cells lining the ventricular system of the developing brain have been identified since the 1960s in animal models and humans (Tennyson and Pappas, 1962; Duckett, 1968) (Figure 1), but Møllgård et al., 1987 was the first to describe the mechanical structure as a “strap” junction using electron microscopy in sheep fetuses. Strap junctions are described as modified tight junctions but differ in both their ultrastructure morphology (Møllgård et al., 1987) and genetic makeup (Whish et al., 2015); however, the function of strap junctions remains similar to tight junctions. Mammalian animal studies have demonstrated that strap junctions also restrict the passage of molecules from the CSF to the brain tissue (Fossan et al., 1985; Whish et al., 2015). Interestingly, the permeability of the barrier appears to become less restrictive over the course of development. Whish et al. (2015) demonstrated molecule diffusion across the barrier in mice embryos was restricted against the smallest molecules (286 Da). The permeability appeared to increase with age in the mice of the same study, with noted extravasation of the author’s largest tracer (70 kDa) in the adult (Whish et al., 2015). The authors noted that the increase in CSF-brain permeability correlate with the disappearance of the strap junctions at later stages of development. As animals age, strap junctions are replaced by gap and adherens junctions (Møllgård et al., 1987), but it is not clear when this switch takes place. One limitation of the study is the limited number of animals used, with one time point only having one representative pup, and lack of diversification of litters. Notwithstanding, the current evidence suggests that the fetal brain may be *more* restrictive than the adult brain with respects to the CSF-brain barrier.

### Comparing the barriers

It is clear that the barriers are important for protecting the brain and maintaining its delicate homeostasis in both the developing and mature brain. Considering every brain cell is within approximately 25 µm from a capillary vessel (Abbott et al., 2010), the BBB protects the entirety of the brain parenchyma. In contrast, the responsibilities of the BCSFB lie at the more anatomically restricted location of the brain’s ventricles and at the pial surface. Within the ventricles, both the BCSFB and the CSF-brain barrier exist in the developing brain. The BCSFB prevents substrates from entering the CSF, while the CSF-brain barrier obstructs substrates in the CSF from freely entering cells of the ventricular epithelium. All three barriers mechanically block the passage of substrates through either their tight junctions (BBB, BCSFB) or strap junctions (CSF-brain). The BBB and BCSFB also have the ability to efflux compounds back into the bloodstream. The BCSFB shares many of the same transporters as the BBB (Redzic, 2011); however, transporter expression at each barrier is distinct (Ek et al., 2010; Morris et al., 2017; Huttunen et al., 2022).

The BBB and BCSFB also have different anatomical locations of their tight junctions. The tight junctions are localized to the luminal surface of the blood vessels of the BBB and the luminal surface of the choroid plexus of the BCSFB. However, the blood vessels invaginating the BCSFB do not contain tight junctions, permitting for the passage of macromolecules into the extracellular space bordering the choroidal epithelium (Brightman, 1968). In a non-disease state, molecules with a diameter of ∼12 nm may diffuse through the capillary pores into the extracellular space (Sarin, 2010). Molecules such as sucrose, inulin, albumin, and IgG do not cross the BBB but can slowly cross the choroid plexus (see Pardridge 2016 for review). As such, BCSFB is considered to exhibit low paracellular permeability in comparison to the BBB that exhibits near zero. The differences in permeability are also attributed to physical processes. Blood flow at the BCSFB is significantly faster than at the BBB, indicating more likely contact with blood-bound substrates. Despite the argument that the BCSFB is considered “leaky,” the BBB is propounded to be the major interface of concern. This is because the capillaries of the BBB come into contact with nearly every brain cell, whereas the CSF only contacts certain brain regions (Figure 1). This key difference is recognized in drug development. Therapeutics that enter the CSF are not often considered fully efficacious as they cannot permeate the brain tissue uniformly, and instead are preferentially delivered to cells that contact the CSF (Pardridge, 2016).

## The developmental ontogeny of the barriers in animal models and humans

To directly compare animal models to humans, we will be using the terminology gestational day (GD) for animals and weeks post conception (WPC) for humans. Therefore, we have adjusted some of the published animal data to standardize that GD 0 indicates the first day of gestation, or the observed sperm positive day for rodents. However, some authors did not define what they considered the sperm positive date (e.g., GD 0, GD 0.5, or GD 1). Therefore, the discussed time points in this section may be within one gestational day for rodents. Any human data that used the terminology of “weeks pregnant” were translated to WPC assuming a 28-day menstrual cycle (e.g., 40 weeks pregnant is 38 weeks post conception). It is also important to note that the patterning of certain anatomical structures varies between species as it most likely relates to the length of gestation and brain growth (Dziegielewska et al., 2001). We will be focusing on data from mice, rats, and humans, which have average gestations of 19 days, 21 days, and 40 weeks, respectively.

Mammalian brain formation begins during embryogenesis, which is closely followed by its vascularization to allow for the tissue to receive the needed oxygen and nutrients. In rodent models, immunohistochemical stains show angiogenesis in the cortical brain structures starting at GD 11 for mice (Sturrock, 1979; Daneman et al., 2009) and GD 12 for rats (Daneman et al., 2010). This process in the human brain is believed to occur between 5 and 7 WPC (Allsopp and Gamble, 1979; Raybaud, 2010; Møllgård et al., 2017) as evidenced by immunohistochemical (Møllgård et al., 2017) and light and electron microscopy observations (Allsopp and Gamble, 1979). The choroid plexus develops after vascularization, and can be seen as early as GD 11 in mouse (Sturrock, 1979; Lun et al., 2015), GD 12 in rats (Dziegielewska et al., 2001), and 7 WPC in humans (Dziegielewska et al., 2001; Møllgård et al., 2017). These developmental time points remain fairly consistent throughout the literature; however, the formation and functionality of the barrier systems remains a point of contention between environmental toxicology and basic science.

The morphological basis of the barriers occurs at the level of cell junction complexes known as tight junctions (see “Cellular properties”). Immunohistochemical studies have suggested that BBB endothelial cells express tight junction proteins (e.g., occludin, claudin-5, and ZO-1) on the same day that angiogenesis begins in rats and mice (Daneman et al., 2010). It also appears that tight junctions are present as soon as blood vessels penetrate the human embryonic brain (Møllgård et al., 2017). Ultrastructural studies have also shown tight junctions in cerebral endothelial cells at the earliest age studied in mice (Bauer et al., 1993), rats (Donahue and Pappas, 1961), and humans (Møllgård and Saunders, 1975, 1986). Microscopy studies have repeatedly demonstrated that the developing brain barriers appear similar to the adult in multiple species (Bohr and Møllgård, 1974; Bradbury, 1979; Bauer et al., 1993; Bertossi et al., 1999; Virgintino et al., 2004). Additionally, efflux transporters (e.g., p-glycoprotein, breast cancer resistant protein, and multidrug resistant proteins), another critical component of barrier function, are detected via immunohistochemical staining and/or gene expression as early as GD 10.5–13 in mice (Qin and Sato, 1995; Tachikawa et al., 2005; Ek et al., 2010), GD 13 in rats (Daneman et al., 2010; Ek et al., 2010), and 5 WPC in humans (Møllgård et al., 2017). It should be noted that the expression of these transporters changes throughout development and are not identical to the adult. In some instances, the embryo/fetus will have higher expression of certain efflux proteins or upregulation of genes associated with tight junctions compared to the adult (Liddelow et al., 2012; Saunders et al., 2019).

Similar to the BBB, the BCSFB also appears to have function at the time of formation, suggesting that there is *not* a time period in which the embryo’s brain is completely susceptible to xenobiotics. The choroid plexus forms within the cerebral ventricles (see Dziegielewska et al., 2001 for review), with tight junctions present early in development. Ultrastructure studies have also demonstrated that tight junctions are morphologically similar to adults at the earliest stage studied in rats (GD 14) (Tauc et al., 1984) and humans (7 WPC) (Møllgård and Saunders, 1986). Limited ultrastructure studies have directly looked at tight junctions in mouse embryos; however, it appears that tight junctions are found on differentiating choroid plexus cells across mammalian species (Møllgård and Saunders, 1986; Ek et al., 2003). Efflux transporters are also detected in human (8 WPC) and rat (GD 15) embryos as evidenced by immunostaining (Møllgård et al., 2017) and transcriptomics (Kratzer et al., 2013). Similar results have also been demonstrated in mice (Liddelow et al., 2012).

Structure alone is insufficient to demonstrate a functionally adequate barrier. In support of the aforementioned data, the use of tracers has also signaled that the fetal barrier systems are indeed established during development. However, early tracer studies underwent the same scientifically insidious problem as some of the early dye studies (Behnsen, 1926, 1927; Penta, 1932; Saunders et al., 2015): injection of excessive volumes resulted in extravasation of the dye in the brain tissue, resulting in an inaccurate result that led to the conclusion of a leaky barrier. This experimental artifact was most likely due to toxicity and/or physically damaging the cerebral vessels with high injection volume (see Saunders et al., 2015 for review). However, animal models have found functional effectiveness of the nascent barriers. For example, both the BBB and BCSFB appear functional as early as GD 14 in the rat fetus against tracers like colloidal iron (Shimoda, 1963) and horseradish peroxidase (Tauc et al., 1984), respectively. Prior to the 1970s, the use of radioactively labeled tracers (e.g., sucrose, ^32^P, amino acids, proteins, *etc*.) did not accurately demonstrate BBB/BCSFB permeability as it was hard to distinguish between functionally deficient barriers and increased metabolism in the developing brain. It has since been established that metabolically important compounds are consumed at a greater rate than in the adult as opposed to the previously presumed “leakiness.” The more recent use of biotin-labeled small molecules suggest that the barriers are functioning nearly as soon as the brain is vascularized in both mice and rats (Daneman et al., 2010). Although the current discussion has been in mice and rats, it is worth mentioning that studies in the marsupial opossum, a species born at an early stage of brain development, has also demonstrated the obstruction of small molecules across the developmental barriers (Ek et al., 2001; Ek et al., 2003; Ek et al., 2006).

Information regarding human fetuses is incredibly limited. Gröntoft (1954) may be the only functional study (Figure 2C), but other immunocytochemical evidence has shown that endogenous compounds are selectively excluded from the brain. Dziegielewska and Saunders (1988) demonstrated that proteins do not easily pass intercellularly. More recently, Møllgård et al. (2017) found that α-fetoprotein, a plasma protein present in human embryonic circulation, was also excluded from the brain tissue as soon as the neural tube closed (5 WPC, Figures 2D–F). The surmounting evidence is suggestive that there is not a time period in which the barrier function turns “on.” Rather, functionality of the barriers appears almost immediate, with both the BBB and BCSFB exhibiting tight junctions, efflux proteins, and the ability to block molecules from crossing into the brain tissue.

## Contaminants of immediate and emerging concern that may cross the brain barriers

In the previous sections, we reviewed the history and importance of the barrier systems and described the numerous experiments that have delineated their form and function. As designing therapeutics that can bypass the brain barriers is a longstanding bottleneck in neuropharmaceutical development (Pardridge, 2007), the BBB and BCSFB have received much attention in pharmacology. In contrast, the field of environmental toxicology has historically exhibited a more tepid regard to brain barrier biology. In the following sections we will evaluate the current data regarding two environmental contaminants of immediate and emerging concern that may enter brain tissue: per- and polyfluorinated substances (PFAS) and bisphenols. The developmental neurotoxicity of these chemicals are reviewed elsewhere (Mariussen, 2012; O'Shaughnessy et al., 2021; Denuziere and Ghersi-Egea, 2022; Welch and Mulligan, 2022), along with other environmental pollutants such as metals and pesticides (Ek et al., 2012).

### Per- and polyfluoroalkyl substances (PFAS)

PFAS are a large family of anthropogenic compounds used in a variety of consumer and manufactured products such as electric or electronic parts, firefighting foams, hydraulic fluids, oil- and water-resistant clothing, and stain repellants (Buck et al., 2011). These chemicals are organofluorine with either partial or fully fluorinated alkyl chains; the strength of the carbon-fluorine bond makes these substances extremely stable. Their resistance to degradation has resulted in environmental ubiquity, and PFAS can be found in soil and water where they have never been used or manufactured. Humans can be exposed to PFAS occupationally, via ingestion of contaminated drinking water and food, and through inhalation or dermal exposure from sources like household dust and after application of household products (D’Hollander et al., 2010). PFAS was first discovered in human sera in 1968 (Taves, 1968) and has since been found in over 99% of human blood samples (Calafat et al., 2019), including umbilical cord blood (Kingsley et al., 2018). The most commonly detected PFAS in human samples include perfluorohexane sulfonic acid (PFHxS), perfluorooctane sulfonic acid (PFOS), perfluorooctanoic acid (PFOA), perfluorononanoic acid (PFNA), perfluorodecanoic acid (PFDA), and perfluoroundecanoic acid (PFUnA) (ATSDR, 2021). These chemicals often have long half-lives in humans, ranging from weeks to decades (ATSDR, 2021), which, combined with persistent exposure throughout the lifespan, further underscores the importance of understanding the potential neurotoxicity of these compounds.

Evaluation of PFAS concentrations in brain tissue has not been extensive, although some studies have identified these compounds within the brain parenchyma since at least the early 2000s (Kannan et al., 2001; Austin et al., 2003; Van de Vijver et al., 2005). This includes human brain tissue (Table 1) (Maestri et al., 2006; Perez et al., 2013; Mamsen et al., 2019). Both wildlife and *in vivo* experiments have claimed that PFAS crosses the adult BBB, as evidenced by quantifiable PFAS concentrations in brain tissue. However, most of these studies did not perform cardiac perfusion or a similar technique to appropriately exsanguinate organs of blood contamination. As discussed in this review, the brain is highly vascularized (Kirst et al., 2020). Therefore, failure to remove internal and/or residual blood should not be considered a “nonissue” (Greaves et al., 2013), especially when examining chemicals like PFAS which bind to blood proteins (e.g., albumin) (Forsthuber et al., 2020). For example, non-perfused brains from human, wildlife, and *in vivo* studies have shown detectable levels of PFOA. Interestingly, saline perfused brains of adult rats dosed with a large amount of PFOA (50 mg/kg/single dose, oral) did *not* have detectable concentrations within the brain tissue, while the average blood concentration in the exposed rats was much higher than, or comparable to, other *in vivo* data (Kawabata et al., 2017). To date, there have only been two studies that perfused animals prior to brain extraction and analysis (Lau et al., 2006; Kawabata et al., 2017). Lau et al. (2006) reported findings via a conference abstract, while Kawabata et al. (2017) showed that, similar to PFOA, PFDA brain concentrations in adult rats were <1/10 of serum concentrations following a single oral dose. However, perfluorodecanoic acid (PFDoA) brain concentrations were *higher* than the serum, even after saline perfusion to exsanguinate organs. This suggests that some PFAS may enter the brain better than others. Interestingly, PFDoA has a higher molecular weight (614 Da) than either PFOA (414 Da) or PFDA (514 Da), which implies that these differences are likely not due to simple diffusion. This is in contrast to other studies, where authors speculated that small PFAS diffuse across the brain barriers (Greaves et al., 2013; Pizzurro et al., 2019). In addition to the aforementioned experiments, some have attempted to demonstrate the distribution of PFAS in different brain regions (Austin et al., 2003; Eggers Pedersen et al., 2015). But again, these studies do not consider blood contamination and data interpretation is difficult. Blood vessel, diameter and density varies throughout the brain (Zhang et al., 2019), suggesting that some regions may appear to have higher concentrations of PFAS simply because there is more blood contamination in that anatomical area. Another consideration is that the inner regions of the brain (e.g., pons/medulla, hypothalamus, thalamus) are closest to incoming blood flow, placing those areas in contact with the highest xenobiotic concentrations if these compounds can indeed cross the BBB.

**TABLE 1:** Mean (range) concentrations of per- and polyfluorinated substances (PFAS) in human brain and sera samples.

		PFHxS		PFOS		PFOA		PFNA		PFDA		PFUnA	
Reference	Age	Serum	Brain	Serum	Brain	Serum	Brain	Serum	Brain	Serum	Brain	Serum	Brain
Perez et al. (2013)	Adult	NM	3.20 (<LLOQ - 14.4)	NM	4.9 (<LLOQ - 22.5)	NM	<LLOQ	NM	29.7 (<LLOQ - 150)	NM	23.4 (<LLOQ - 204)	NM	<LLOQ
Maestri et al. (2006)	Adult	NM	NM	5.1	1.3	3	0.5	NM	NM	NM	NM	NM	NM
Mamsen et al. (2019)	Fetus, First Trimester	NM	NM	8.14 (2.49–16.66)^a^	<LLOQ	2.04 (0.55–7.95)^a^	0.17 (0.16–0.18)	1.04 (0.41–2.9)^a^	0.10 (0.10–0.10)	0.34 (0.13–0.94)^a^	0.13 (0.13–0.13)	0.46 (0.18–1.73)^a^	<LLOQ
	Fetus, Second Trimester	0.52 (0.08–2.77)^a^	0.70 (0.70–0.70)	3.87 (1.04–8.19)^a^	0.51 (0.23–0.99)	1.62 (0.72–3.78)^a^	0.38 (0.18–0.88)	0.51 (0.19–1.03)^a^	<LLOQ	0.26 (0.07–0.56)^a^	<LLOQ	0.34 (0.11–0.77)^a^	<LLOQ
	Fetus, Third Trimester	0.75 (0.09–5.23)^a^	<LLOQ	3.58 (1.07–9.66)^a^	0.36 (0.19–0.69)	1.62 (0.62–4.62)^a^	0.19 (0.19–0.19)	0.53 (0.14–1.8)^a^	0.12 (0.12–0.12)	0.27 (0.07–1.11)^a^	<LLOQ	0.27 (0.10–0.56)^a^	<LLOQ
													
		Serum	CSF	Serum	CSF	Serum	CSF	Serum	CSF	Serum	CSF	Serum	CSF
Harada et al. (2007)	Adult	NM	NM	17.9 (7.4–31.4)	0.12 (0.07–0.20)	3.7 (2.0–6.3)	0.06 (<LLOQ - 0.07)	NM	NM	NM	NM	NM	NM
Wang et al. (2018)	Adult	0.82 (0.03–13.33)	0.01 (<LLOQ - 0.32)	6.78 (0.17–69.78)	0.03 (<LLOQ - 1.48)	7.44 (0.14–240.47)	0.08 (<LLOQ - 3.00)	1.66 (0.00–10.61)	0.01 (<LLOQ - 0.27)	1.44 (0.04–14.34)	0.01 (<LLOQ - 0.25)	0.92 (0.09–6.71)	0.008 (<LLOQ - 0.13)
Liu et al. (2022)	Neonates (≤28 days)	0.67 (0.09–1.63)	0.01 (0.00–0.09)	2.77 (0.63–7.74)	0.07 (0.01–0.30)	11.3 (2.78–27.1)	0.31 (0.13–0.63)	0.42 (<LLOQ - 1.49)	<LLOQ (<LLOQ - 0.22)	0.74 (0.06–2.50)	0.02 (<LLOQ - 0.13)	0.47 (<LLOQ – 1.86)	<LLOQ
													

Serum and cerebrospinal fluid are reported in ng/ml, except for Maestri et al. (2006) who reported ng/g; brain concentrations are reported in ng/g wet weight. Wang et al., reported geometric mean. The data reported for Liu et al., 2022 are means obtained from 22 neonatal CSF samples and 49 neonatal serum samples, 9 of which were paired. Only paired samples were used to calculate R_CSF/Serum_, as described in the text. CSF, cerebrospinal fluid; LLOQ, lower limit of quantification; NM, not measured; PFHxS, perfluorohexane sulfonic acid; PFOS, perfluorooctanesulfonic acid; PFOA, perfluorooctanoic acid; PFNA, perfluorononanoic acid; PFDA, perfluorodecanoic acid; PFUnA, perfluoroundecanoic acid.

a Maternal serum measurements; fetal serum not reported.

There are several publications that have investigated PFAS in the human central nervous system. Two studies have documented PFAS in adult human CSF (Harada et al., 2007; Wang et al., 2018). Both Harada et al. (2007) and Wang et al. (2018) show that PFAS concentrations in the CSF were about 1% of those in serum, suggesting that these chemicals are not able to freely pass the BCSFB (see Table 1). However, both studies received CSF samples from patients that may have been afflicted with illnesses that could alter the integrity of either the BBB or BCSFB, which could result in increased levels of PFAS in the CSF. Thus, it is possible that levels are even lower in the general population. Limited developmental exposure studies exist in humans. Similar to reports by Harada and Wang, a new study investigated nine paired serum and CSF samples from human neonates (Liu et al., 2022). The authors show in human patients no older than 4 weeks old, the mean CSF:serum ratio (R_CSF/Serum_) of 32 different PFAS was never greater than 0.033 (i.e., ∼3%), and most PFAS in the CSF were below the lower limit of quantification (Liu et al., 2022, Table 1). It is important to note that lumbar punctures are not performed in newborns unless significant health concerns exist (Coughlan et al., 2021). These babies were likely suffering from an illness or disease that warranted this invasive procedure, although the precise disease state for individuals was not disclosed in the study design (Liu et al., 2022). While illness or developmental issues can cause brain barrier dysfunction, and CSF turnover is normally lower in the fetus/infant (see section 3.2 on sink effect), these babies still exhibited a relatively low R_CSF/Serum_. This indicates that PFAS are not freely entering the newborn CSF, although some (specifically linear isomers of PFHxS, PFOS, and PFOA) were still detectable (Liu et al., 2022). In another study, Mamsen et al. (2019) showed higher average levels of PFAS (PFHxS, PFOS, PFOA, PFNA, PFDA, PFUnA) in placenta as compared to the fetal CNS (first trimester samples consisted of spinal cord, second and third trimester samples consisted of brain), suggesting that the brain barriers are functioning. For example, all second and third trimester placentas had detectable levels of PFOS, while only half of the brain samples from these time periods had concentrations above the limit of detection. Mamsen and others also showed that their fetal CNS samples had the lowest PFAS burden of *any* fetal tissues sampled across all trimesters (Mamsen et al., 2019). The latter data are a sound indicator that the brain barriers are actively occluding these PFAS to at least some extent in the human fetus. In comparison to the adult brain concentrations reported in Perez et al. (2013) and Maestri et al. (2006), the average fetal brain PFAS concentrations were always less. However, paired PFAS concentrations in the blood and brain were not reported in the fetus (Mamsen et al., 2019), so it is not possible to make definitive conclusions regarding chemical transfer efficacy in the fetus versus adult. It is important to note that the fetal tissues in Mamsen et al. (2019) were obtained from both elective and spontaneous abortions, so it is possible that some of the fetuses suffered from major birth defects and/or abnormalities that could have affected brain barrier function. This could explain why some fetal CNS tissue had higher levels of PFAS while other samples did not.

Despite the identification of several PFAS compounds in both the adult and developing human brain, current *in vivo* developmental studies are limited and have utilized chickens (Cassone et al., 2012), mice (Borg et al., 2010), and rats (Lau et al., 2006; Chang et al., 2009; Wang et al., 2010; Zeng et al., 2011; Ishida et al., 2017) as model systems. Cassone et al. (2012) detected PFHxS in the cerebral cortex of chicken embryos, but there were no comments on the mechanism of PFHxS transport. The remaining developmental studies primarily focus on the eight carbon PFAS congeners (PFOA, PFOS) in a murine model (Tables 2, 3). From the published data, only Lau et al. (2006) quantified PFOS in saline perfused rat brain tissue after gestational exposure and found that pups had substantially higher levels as compared to the dams, despite dam blood concentrations being over double that of the fetus. This may suggest that PFOS enters the brain more readily in developing animals, but as discussed below, this may not be the case. Ishida et al. (2017) also measured PFOS concentrations in both dams and neonatal pups after gestational exposure but found similar serum concentrations in the adult and developing animals; however, the authors also detected significantly higher PFOS concentrations in the non-perfused pup brain as compared to adults. Chang et al. (2009), Wang et al. (2010), Macon et al. (2011), and Zeng et al. (2011) evaluated either PFOA or PFOS levels at different developmental time points in rats after gestational exposure and noted that brain concentrations decreased with age; however, authors did not comment on the synchronous decrease in blood levels. One postnatal study conducted by Liu et al. (2009) administered one large subcutaneous dose of PFOS (50 mg/kg) to young mice at different developmental time points and found comparable blood PFOS concentrations across the developmental stages tested. In contrast, the brain PFOS concentrations decreased as the mice aged, which could be explained by increased penetration of PFOS in young animals, or alternatively by a different hypothesis. Irrespective of the study, all authors attributed the PFOS concentrations in the young brains to an “incomplete,” “immature,” “porous,” and/or “undeveloped” BBB (Table 4). There are several possibilities for the observed trending decrease in cerebral PFAS concentrations in rodents. Borg et al. (2010) investigated heavy labeled PFOS distribution in the fetal rat and remarked uneven signal in the brain. The authors commented that PFOS did not appear concentrated in the fetal cortex, and their published autoradiograms instead show PFOS amassed in the ventricles. This is an interesting observation as the developing brain has a much lower turnover of CSF. The slow turnover rate results in slower clearance, and thus a greater accumulation, of compounds in the CSF as compared to older animals (Saunders et al., 2014). Relative to brain size, the developing brain also has much larger ventricles which could contribute to the higher levels of PFAS observed. Finally, the young postnatal brain also undergoes rapid expansion known as the brain growth spurt, and this occurs in both rodents and humans. Peak brain growth in rats occurs a week after birth (Dobbing and Sands, 1979). This corresponds to the observed decreasing level of PFAS in these aforementioned studies (Chang et al., 2009; Wang et al., 2010; Macon et al., 2011; Zeng et al., 2011), which express PFAS concentrations as nanogram of chemical per gram of brain parenchyma. In all, although some publications do report large amounts of PFAS in the brain, these studies are confounded by several technical variables. In humans and animal models that study either environmentally relevant PFAS levels and/or control for variables like blood contamination, it appears that the brain barriers occlude many of these chemicals to varying degrees.

**TABLE 2:** Pregnant laboratory mice were exposed to perfluorooctanoic acid (PFOA) daily through different exposure routes, doses, and durations as provided below.

Reference	Species (N)	Route of exposure	Dose (mg/kg)	Duration (days)<	Developmental stage for tissue collection	Serum (µg/ml)	Brain (µg/g ww)	Perfused brain?
Macon et al. (2011)	Mouse (4)	Gavage	0.3	GD1 - GD17	Neonatal, female (PN6)	4.980 ± 0.218	0.150 ± 0.026	No
	Mouse (6)	Gavage	0.3	GD1 - GD17	Juvenile, female (PN13)	4.535 ± 0.920	0.065 ± 0.012	No
	Mouse (5)	Gavage	0.3	GD1 - GD17	Juvenile, female (PN20)	1.194 ± 0.394	< LLOQ	No
	Mouse (5)	Gavage	1.0	GD1 - GD17	Neonatal, female (PN6)	11.026 ± 0.915	0.479 ± 0.041	No
	Mouse (6)	Gavage	1.0	GD1 - GD17	Juvenile, female (PN13)	16.950 ± 3.606	0.241 ± 0.020	No
	Mouse (5)	Gavage	1.0	GD1 - GD17	Juvenile, female (PN20)	3.770 ± 0.607	0.031 ± 0.005	No
	Mouse (2–4)	Gavage	3.0	GD1 - GD17	Neonatal, female (PN6)	20.700 ± 3.900	1.594 ± 0.162	No
	Mouse (4)	Gavage	3.0	GD1 - GD17	Juvenile, female (PN13)	26.525 ± 2.446	0.650 ± 0.044	No
	Mouse (3)	Gavage	3.0	GD1 - GD17	Juvenile, female (PN20)	8.343 ± 1.078	0.133 ± 0.023	No
Onishchenko et al. (2011)	Mouse (4)	Diet	0.3	GD0 - GD21	Neonatal (PN0)	NM	0.7 ± 0.1	No

PFOA concentrations were quantified in the sera and brains of mouse pups (postnatal day 0 – postnatal day 20). Abbreviations: GD, gestational day; LLOQ, lower limit of quantification; NM, not measured; PN, postnatal day.

**TABLE 3:** Pregnant and developing laboratory animals were exposed to perfluorooctanesulfonic acid (PFOS) through different exposure routes, doses, and durations as provided below.

Reference	Species (N)	Route of exposure	Dose (mg/kg)	Duration (days)	Developmental stage for tissue collection	Serum (µg/ml)	Brain (µg/g ww)	Perfused brain?
Borg et al. (2010)	Mice (9)	Gavage/IV	12.5	GD15	Fetus (GD17)	∼18	∼11	No
	Mice (7)	Gavage/IV	12.5	GD15	Fetus (GD19)	∼9	∼8	No
	Mice (6)	Gavage/IV	12.5	GD15	Neonatal (PN0)	∼14	∼8	No
Chang et al. (2009)	Rat (≤10)	Gavage^a^	0.1	GD0 - GD19	Fetus (GD20)	3.91 ± 0.10	1.23 ± 0.07	No
	Rat (≤25)	Gavage^a^	0.1	GD0 - PN3	Neonatal (PN4)	2.24 ± 0.07	0.68 ± 0.03	No
	Rat (≤25)	Gavage^a^	0.1	GD0 - PN20	Juvenile, Male (PN21)	1.73 ± 0.08	0.22 ± 0.01	No
	Rat (≤25)	Gavage^a^	0.1	GD0 - PN20	Juvenile, Female (PN21)	1.77 ± 0.08	0.23 ± 0.01	No
	Rat (≤10)	Gavage^a^	0.3	GD0 - GD19	Fetus (GD20)	10.45 ± 0.29	3.17 ± 0.24	No
	Rat (≤25)	Gavage^a^	0.3	GD0 - PN3	Neonatal (PN4)	6.96 ± 0.16	1.91 ± 0.07	No
	Rat (≤25)	Gavage^a^	0.3	GD0 - PN20	Juvenile, Male (PN21)	5.05 ± 0.11	0.65 ± 0.05	No
	Rat (≤25)	Gavage^a^	0.3	GD0 - PN20	Juvenile, Female (PN21)	5.25 ± 0.14	0.73 ± 0.04	No
	Rat (≤10)	Gavage^a^	1.0	GD0 - GD19	Fetus (GD20)	31.46 ± 1.03	12.98 ± 1.12	No
	Rat (≤25)	Gavage^a^	1.0	GD0 - PN3	Neonatal (PN4)	22.44 ± 0.72	6.68 ± 0.43	No
	Rat (≤25)	Gavage^a^	1.0	GD0 - PN20	Juvenile, Male (PN21)	18.61 ± 1.01	2.62 ± 0.17	No
	Rat (≤25)	Gavage^a^	1.0	GD0 - PN20	Juvenile, Female (PN21)	18.01 ± 0.74	2.70 ± 0.19	No
Ishida et al. (2017)	Rat (8–27)	Gavage	1.0	GD11 - GD20	Neonatal (PN4)	19.3 ± 0.51	8.4 ± 0.4	No
	Rat (8–27)	Gavage	2.0	GD11 - GD20	Neonatal (PN4)	37.2 ± 0.98	15.1 ± 0.5	No
Lau et al. (2006)	Rat (NA)	Gavage	3	GD2 - GD21	Neonatal (PN7)	52	16–29	Yes
Liu et al. (2009)	Mouse (4–6)	Subcutaneous injection^b^	50	PN6	Neonatal (PN6)	∼90	∼50	No
	Mouse (4–6)	Subcutaneous injection^b^	50	PN13	Juvenile (PN13)	∼95	∼45	No
	Mouse (4–6)	Subcutaneous injection^b^	50	PN20	Juvenile (PN20)	∼80	∼45	No
	Mouse (4–6)	Subcutaneous injection^b^	50	PN27	Juvenile (PN27)	∼80	∼20	No
	Mouse (4–6)	Subcutaneous injection^b^	50	PN34	Juvenile (PN34)	∼95	∼30	No
Onishchenko et al. (2011)	Mouse (4)	Diet	0.3	GD0 - GD21	Neonatal (PN0)	NS	3.1 ± 0.3	No
Wang et al. (2010)	Rat (≤10)	Diet	3.2	GD0 - PN0	Neonatal (PN0)	5.978 ± 0.514	2.085 ± 0.108	No
	Rat (≤10)	Diet	3.2	GD0 - PN6	Neonatal (PN6)	NM	1.516 ± 0.085	No
	Rat (≤10)	Diet	3.2	GD0 - PN13	Juvenile (PN13)	NM	1.416 ± 0.083	No
	Rat (≤10)	Diet	3.2	GD0 - PN20	Juvenile (PN20)	8.9125 ± 0.106	0.974 ± 0.062	No
	Rat (≤10)	Diet	3.2	GD0 - PN20	Juvenile (PN34)	11.0775 ± 0.369	0.588 ± 0.028	No
Zeng et al. (2011)	Rat (5)	Gavage	0.1	GD2 - GD21	Neonatal (PN0)	1.50 ± 0.43	0.39 ± 0.09	No
	Rat (6)	Gavage	0.1	GD2 - GD21	Juvenile (PN21)	0.37 ± 0.12	0.06 ± 0.04	No
	Rat (5)	Gavage	0.6	GD2 - GD21	Neonatal (PN0)	24.60 ± 3.02	5.23 ± 1.58	No
	Rat (6)	Gavage	0.6	GD2 - GD21	Juvenile (PN21)	1.86 ± 0.35	1.03 ± 0.59	No
	Rat (5)	Gavage	2.0	GD2 - GD21	Neonatal (PN0)	45.69 ± 4.77	13.43 ± 3.89	No
	Rat (6)	Gavage	2.0	GD2 - GD21	Juvenile (PN21)	4.26 ± 1.73	3.69 ± 0.95	No

Only one study (Liu et al., 2009) directly dosed young animals with PFOS; otherwise, only maternal animals were dosed. A range of duration is indicative of daily dosing; a single date indicates one dose. PFOS concentrations were then quantified in the serum and brain of developing laboratory animals. Abbreviations: GD, gestational day; LLOQ, lower limit of quantification; NM, not measured; PN, postnatal day.

a Route of exposure not explicitly stated by the authors.

b Direct dosing of pups performed.

**TABLE 4:** Statements regarding an immature blood-brain barrier as reasoning for perfluorooctanoic acid (PFOA) or perfluorooctanesulfonic acid (PFOS) crossing into the developing brain with comments regarding the unsupported evidence.

Reference	Quotation and comments
Lau et al. (2006)	“*…substantially higher concentrations of PFOS were detected in the neonatal rat brain, likely due to incomplete formation of the blood-brain barrier at that developmental stage.”* Abstract/poster presentation. No supporting evidence provided.
Chang et al. (2009)	“…*these data suggest that brain uptake may have been higher in fetal rats, perhaps due to the undeveloped state of the “blood–brain barrier” on GD 20… In rats, the “blood–brain barrier” is not fully developed until PND 24, hence embryonic and early neonatal brain capillaries are more permeable for substances* (Kniesal et al., 1996).” see Saunders et al. (2014): “did not include any permeability studies in parallel with their ultrastructural observations. Instead, they relied on comparisons with *in vitro* cultures of cerebral endothelial cells to support their conclusion that age correlated with supposed greater blood–brain barrier permeability in the developing brain.”
Liu et al. (2009)	“*The variation in the distribution of PFOS in the mice observed may be formed accompanying the development process which mainly includes the establishment and ripeness of blood-brain barrier function* (Watson et al., 2006) …” The Watson et al. (2006) review uses many of the same sources that Saunders et al. (2014) and Ek et al. (2012) refute. Cross references.
Borg et al. (2010)	*“This difference is presumably due to the incomplete development of the fetal blood–brain barrier* (Lau et al., 2006; Chang et al., 2009) *…”* Neither paper provides evidence for barrier immaturity.
Wang et al. (2010)	*“Because the BBB in neonatal rats is quite immature and porous at birth and is not completed until PND 24* (Chang et al., 2009 *) … PND 35 is regarded as a time when the brain is mature.*” Chang et al. (2009) does not provide evidence for barrier immaturity. No other supporting evidence provided.
Macon et al. (2011)	“*The presence of PFOA in the neonatal brain, coupled with its absence after 4 weeks of age, suggests that PFOA passes through the fetal mouse blood-brain barrier but is not able to pass through the fully functional barrier that is normally formed by the time of birth* (Bauer et al., 1993).” Bauer et al. (1993) data supports the idea that BBB properties are present prior to birth; this was an incorrect citation for this statement.
Onishchenko et al. (2011)	“*Analyses of human and animal samples have shown that PFOS accumulates in the developing brain before formation of the blood–brain barrier (BBB) but can also cross mature BBB to a certain extent (* *Maestri et al., 2006*; *Harada et al., 2007*; *Chang et al., 2009* *).*” Maestri et al. (2006) and Harada et al. (2007) were conducted in adults. Chang et al. (2009) does not provide evidence for barrier immaturity.
Zeng et al. (2011)	*“…the blood–brain barrier in embryonal and neonatal rats is quite immature and porous and is not completed until PND 24* (Chang et al., 2009)*. Immaturity of the BBB lacks the protective effect…”* Chang et al. (2009) does not provide evidence for barrier immaturity.
Ishida et al. (2017)	“*Gestational and lactational exposures to PFOS resulted in brain PFOS concentrations in immature rodents approximately ten times higher than in maternal brain because the BBB in neonatal and fetal animals is not completely established* (Chang et al., 2009).” Chang et al. (2009) does not provide evidence for barrier immaturity.
Pizzurro et al. (2019)	*“…PFOS concentrations in the rat pup hippocampus and cortex decreased between post-natal day (PND) 0 and PND 21, consistent with the fetal blood-brain barrier not being fully developed.*” No supporting evidence.

There is very little information available about the mode of transport for PFAS across the brain barriers, including whether or not the compounds act similarly to one another *in vivo*. There is some speculation that PFAS could diffuse or actively pass through specific membrane transporters (Piekarski et al., 2020), but there are no studies that specifically address either hypothesis. However, the data at hand do not seem to support simple diffusion across the barriers, as the brain and CSF concentrations are much lower than blood levels for those that disseminated paired tissue samples. In addition, studies in animals and humans have shown that brain PFAS concentrations do not seem to correlate to their molecular weight (Kawabata et al., 2017; Wang et al., 2018); if simple diffusion was occuring, smaller PFAS would generally cross into the brain with greater efficacy. Instead, the data at hand suggests the brain barriers are restricting PFAS entry into the brain in developing and adult animals (including humans), but some PFAS are able to bypass the barriers by an unknown mechanism. One potential mechanism for PFAS entry in the adult brain is through alterations of tight junctions, and thus abnormal brain barrier function. Yu et al. (2020) observed decreases in the expression of tight junction-related proteins (ZO-1, claudin-5, claudin-11, occludin) and ultrastructural changes in the BBB in the cerebral cortex of adult mice orally exposed to PFOS. No functional assays were implemented, but their data suggest that PFOS could cross into the tissue through BBB disruption. Wang et al. (2018) showed that in their human samples, high estimates of brain barrier permeability in individuals corresponded to higher PFAS concentrations in CSF. As previously mentioned, these CSF samples were collected via lumbar puncture in adult patients that may have been afflicted with neurological disorders that caused brain barrier dysfunction irrespective of PFAS toxicity. No other studies have investigated BBB disruption in the developing brain. If BBB disruption is occurring, the developing brain could be left more susceptible to interactions with other blood-bound xenobiotics and/or pathogens*.* As there may be permanent neurological consequences due to early life perturbations of the brain barriers (Najjar et al., 2017; Greene et al., 2018; Pollak et al., 2018; Baruah et al., 2019; Medin et al., 2019; Kealy et al., 2020), further mechanistic and functional studies to address whether PFAS can disrupt the brain barriers are warranted.

### Bisphenols

Bisphenols are a vast family of chemicals, but *in vivo* toxicity data exist for only a small subset (KEMI 2017; Pelch et al., 2017; Pelch et al., 2019). They are primarily used to make polycarbonate plastics and epoxy resins. Polycarbonate plastics are often used in products such as single-use beverage containers, reusable containers, tableware, and water pipes; epoxy resins can be used as protective lining in food and beverage cans, metal lids on containers, and dental sealants. Other commercial uses include electronics, floor sealants, medical devices, paints, personal care products, thermal receipts, and toys (Chen et al., 2016; Pelch et al., 2017). Human exposure is thought to primarily occur through diet, but other exposure routes include medical and dental products, and household dust (Vandenberg et al., 2007).

Bisphenol A (BPA) is the best studied bisphenol, and one of the most extensively investigated endocrine disrupting chemicals in toxicology. The compound’s estrogenic activity has been recognized since 1936 (Dodds and Lawson, 1936), and there is now evidence to suggest that it also interacts with androgen receptors, sex hormone-binding globulin (SHBG), and thyroid receptors (Mustieles et al., 2015; Vom Saal and Vandenberg, 2021). Growing concerns for BPA’s health effects in humans has resulted in the use of alternative analogues, commonly Bisphenol AF, F, and S (Chen et al., 2016). However, BPA is still often the dominant bisphenol detected in both abiotic and biological matrices (Chen et al., 2016). Production and usage data for bisphenols around the world is generally lacking (Chen et al., 2016), but it appears as though BPA is also one of the most produced chemicals in the world per year (OECD, 2009). Although BPA has been used in plastics since the 1950s (Vom Saal and Vandenberg, 2021) and known to leach from plastics since at least the early 1990s (Krishnan et al., 1993), human biomonitoring studies measuring BPA was not documented until 2005 (Calafat et al., 2005). Now, more than 90% of European and American urine samples have detectable concentrations of BPA (Mustieles and Fernandez, 2020). Studies have also measured this chemical in other human tissues, including blood and amniotic fluid (Ikezuki et al., 2002), but the published data has been under scrutiny as biomonitoring and kinetic studies have reached conflicting conclusions (Vandenberg et al., 2013; Vom Saal and Vandenberg, 2021). Every oral human pharmacokinetic study has shown that the half-life of total BPA (free and conjugated) in adult humans is approximately 6 h (Volkel et al., 2002; Thayer et al., 2015) and nearly 100% is eliminated through the urine within 24 h (Volkel et al., 2002; Volkel et al., 2005; Teeguarden et al., 2015; Thayer et al., 2015). More recently, Sasso et al. (2020) found the half-life of BPA via dermal exposure was slightly longer at approximately 20 h. Because humans can metabolize BPA rapidly, it is speculated that the high human exposure estimates (i.e., within the ng/ml range) may be erroneous, due to BPA contamination from the laboratory plastics used to collect, store, and process biological samples (Doerge et al., 2011; Vandenberg et al., 2013). Although plastic contamination is a potential issue that could artificially inflate exposure estimates, an analysis of CDC data from the National Health and Nutrition Examination Survey proposed that the half-life of BPA may be longer than expected, and humans are ubiquitously exposed via multiple routes (Stahlhut et al., 2009). This suggests that the wide human exposure ranges may not be entirely inaccurate and result from variations in exposure due to differing lifestyle choices.

Extensive literature (500+ articles) investigates the implications of BPA exposure on the brain (Patisaul, 2020), but very few studies evaluate BPA concentrations in the brain, the mechanism by which it may cross the brain barriers, and/or whether the observed endpoints are a result of BPA indirectly interacting with the brain (e.g., affecting the brain through an endocrine or inflammatory mediated mechanism). Corrales et al. (2015) compiled a total of 63 wildlife studies since 1999 that reported BPA concentrations in wildlife (mainly fish, invertebrates, and amphibians), and only one of the studies included chemical estimates in the brain (Renz et al., 2013). Since the 2015 compilation, other wildlife studies have documented the concentrations of bisphenols in the brains of different bird species (Gonzalez-Rubio et al., 2020; Bodziach et al., 2021) and fish (Ros et al., 2016). All three studies failed to provide for bisphenol serum concentrations, making it difficult to draw definitive conclusions regarding transfer across the BBB. Renz et al. (2013) posited that chemicals may be entering the fish brain by either crossing the BBB or via axonal transport. Bodziach et al. (2021) mentioned that the waterbird brains appeared protected from some of the bisphenols measured, but the authors did not speculate on the seemingly large observed BPA concentrations.

*In vivo* work in rats, mice, and monkeys has also shown BPA in brain tissue, but it has not been particularly consistent (Table 5). In addition to different animal models, studies vary by dosing scheme, the BPA analytes measured (aglycone, conjugated, or total), the brain compartment evaluated, and the time between the last administered BPA dose and tissue analysis. These vastly different experimental designs make it exceedingly difficult to compare results. For example, Shin et al. (2004) reported BPA-brain concentrations at almost double serum concentration levels in adult male rats given an intravenous injection (0.5 mg/kg) every 30 min for 4.5 h, whereas Yoo et al. (2000) found BPA-brain concentrations to be less than serum levels in adult male rats administered a simultaneous intravenous bolus injection (0.73 mg/kg) and infusion to steady state (0.5 mg/h). Neither study speculated on how the chemical reached relatively high concentrations in the brain. Kim et al. (2004) found that brain tissue concentration increased linearly with the oral dose and that there were comparable levels of BPA concentrations in seven different brain regions in the rat 48 h after oral dosing. The authors interpreted these results to mean that BPA can “penetrate [the brain] freely” (Kim et al., 2004). The authors did not mention their possibility of blood contamination in the brain from the lack of perfusion.

**TABLE 5:** Concentrations of bisphenols in human brain.

Reference	Age	Congener	Brain region	N (total)	Brain concentration Mean (min-Max)
Geens et al. (2012)	Children/Adult	BPA	Brain sample^a^	8 (11)	0.91 (<LLOQ – 2.36)
van der Meer et al. (2017)	Adult	BPA	Hypothalamus	23 (24)	3.17 ± 6.42 (0.32–26.62)
	Adult	BPA	White matter tract	9 (24)	1.23 ± 1.07 (0.30–3.32)
Charisiadis et al. (2018)	Adult	BPA	Hypothalamus	23 (24)	2.52 ± 2.95 (0.9–14.5)
	Adult	BPA	“Fresh” hypothalamus^b^	3 (3)	2.67 ± 0.49 (2.1–3.0)
	Adult	BPA	White matter tract	12 (12)	1.65 ± 1.37 (0.9–5.7)
	Adult	BPF	Hypothalamus	23 (24)	3.95 ± 6.06 (1.7–30.8)
	Adult	BPF	“Fresh” hypothalamus^b^	3 (3)	5.57 ± 1.94 (3.9–7.7)
	Adult	BPF	White matter tract	12 (12)	2.40 ± 0.51 (1.7–3.4)

No corresponding serum samples were collected. Mean (range) concentrations are reported in ng/g wet weight. BPA, bisphenol A; BPF, bisphenol F; LLOQ, lower limit of quantification.

a Brain region not specified.

b Fresh defined as samples collected within 24-h postmortem.

Consistent with the wildlife and *in vivo* studies, human studies show detectable levels of BPA in adult brain tissue. Geens et al. (2012), van der Meer et al. (2017), and Charisiadis et al. (2018) detected BPA in more than 70% of brain samples collected from postmortem adult humans at concentrations ranging from <0.4–26.62 ng/g. All studies reported relatively similar concentrations with median values of 0.57, 0.68 and 1.2 ng/g respectively (see Table 6). Charisiadis et al. (2018) also found almost double median BPF concentrations compared to BPA. van der Meer et al. (2017) and Charisiadis et al. (2018) determined that there was not any preferential accumulation of BPA or BPF between two different brain regions (hypothalamus and white matter tract), which the authors deemed suggestive of the chemicals crossing the BBB. The authors did not propose a potential mechanism for the compounds crossing, nor did they speculate about the potential contamination from residual blood in the brain tissue.

**TABLE 6:** Concentrations of bisphenol A (BPA) in the serum and brain of exposed laboratory animals.

Reference	Species (N)	Route of exposure	Dose	Developmental stage for tissue collection	Serum	Brain	Brain region	Perfused brain?
Yoo et al. (2000)	Rat (4)	Intravenous	0.73 mg/kg + iv 0.5 mg/h	Adult	314.9 ± 86.6 ng/ml	236.0 ± 145.5 ng/g	Whole	No
Nunez et al. (2001)	Rat (3)	Subcutaneous implant	1 mg/day	Adult	∼210 ng/ml	137 ng/g	Hypothalamus	No
	Rat (3)	Subcutaneous implant	5 mg/day	Adult	∼290 ng/ml	∼290 ng/g	Hypothalamus	No
Uchida et al. (2002)	Monkey (2)	Subcutaneous	50 mg/kg	Fetal (GD150)	1.70 μg/ml	52.50 μg/g	Cerebrum	No
	Mouse (3–5)	Subcutaneous	100 mg/kg	Fetal (GD17)	∼1.5 μg/ml	∼6 μg/g	Whole^a^	No
	Mouse (3–5)	Subcutaneous	100 mg/kg	Adult	∼2.25 μg/ml	∼8 μg/g	Whole^a^	No
Domoradzki et al. (2003)	Rat (4)	Gavage	10 mg/kg	Fetal (GD6-10)	ND	ND	Whole^a^	No
	Rat (4)	Gavage	10 mg/kg	Fetal (GD14-18)	ND	ND	Whole^a^	No
	Rat (4)	Gavage	10 mg/kg	Fetal (GD17-21)	ND	ND	Whole^a^	No
Kim et al. (2004)	Rat (5)	Oral	100 mg/kg	Adult	0.540 ± 0.064 μg/ml	0.745 ± 0.400 μg/g	Pituitary	No
						0.180 ± 0.107 μg/g	Hypothalamus	No
						0.103 ± 0.046 μg/g	Brain stem	No
						0.102 ± 0.026 μg/g	Cerebellum	No
						0.097 ± 0.033 μg/g	Frontal Cortex	No
						0.181 ± 0.075 μg/g	Hippocampus	No
						0.220 ± 0.122 μg/g	Caudate Nucleus	No
Shin et al. (2004)	Rat (4)	Intravenous	0.5 mg/kg	Adult	∼500 ng/g	∼1000 ng/g	Whole	No
Doerge et al. (2011)	Rat (5)	Oral	100 μg/kg	Fetus (GD20)	< LOD = 0.2 nM^b^	< LOD = 0.4 pmol/g^b^	Whole^a^	No
	Rat (5)	Oral	100 μg/kg	Fetus (GD20)	14 pmol/g^c^	1.5 pmol/g^c^	Whole^a^	No
	Rat (4)	Oral	100 μg/kg	Neonatal (PN3)	∼2.5 pmol/g^b^	∼5 pmol/g^b^	Whole^a^	No
	Rat (4)	Oral	100 μg/kg	Juvenile (PN10)	∼1.55 pmol/g^b^	∼1.5 pmol/g^b^	Whole^a^	No
	Rat (4)	Oral	100 μg/kg	Juvenile (PN21)	∼0.6 pmol/g^b^	∼0.5 pmol/g^b^	Whole^a^	No
	Rat (4)	Oral	100 μg/kg	Neonatal (PN3)	∼230 pmol/g	∼12.5 pmol/g	Whole^a^	No
	Rat (4)	Oral	100 μg/kg	Juvenile (PN10)	∼160 pmol/g	∼3 pmol/g	Whole^a^	No
	Rat (4)	Oral	100 μg/kg	Juvenile (PN21)	∼180 pmol/g	∼3 pmol/g	Whole^a^	No
	Rat (7)	Intravenous	100 μg/kg	Adult	11 ± 3.8 pmol/g^b^	28 ± 8.2 pmol/g^b^	Whole^a^	No
	Rat (7)	Intravenous	100 μg/kg	Adult	57 ± 27 pmol/g^c^	1.6 ± 2.1 pmol/g^c^	Whole^a^	No
Mita et al. (2012)	Mouse (∼10)	Subcutaneous	100 μg/kg	Adult, female	NM	∼0.1 ng/g	Forebrain	No
	Mouse (∼10)	Subcutaneous	1000 μg/kg	Adult, female	NM	∼0.15 ng/g	Forebrain	No
	Mouse (∼10)	Subcutaneous	100 μg/kg	Adult, female	NM	∼0.25 ng/g	Hindbrain	No
	Mouse (∼10)	Subcutaneous	1000 μg/kg	Adult, female	NM	∼0.60 ng/g	Hindbrain	No
	Mouse (∼10)	Subcutaneous	100 μg/kg	Adult, male	NM	∼0.25 ng/g	Forebrain	No
	Mouse (∼10)	Subcutaneous	1000 μg/kg	Adult, male	NM	∼0.35 ng/g	Forebrain	No
	Mouse (∼10)	Subcutaneous	100 μg/kg	Adult, male	NM	∼1.45 ng/g	Hindbrain	No
	Mouse (∼10)	Subcutaneous	1000 μg/kg	Adult, male	NM	∼1.1 ng/g	Hindbrain	No
	Mouse (∼10)	Subcutaneous	100 μg/kg	Adult, male	NM	∼0.1 ng/g	Forebrain	No
	Mouse (∼10)	Subcutaneous	1000 μg/kg	Adult, male	NM	∼0.15 ng/g	Forebrain	No
Patterson et al. (2013)	Monkey	Intravenous	100 μg/kg	Fetal (3)	1.4 ± 0.56^d^ pmol/g	1.3 pmol/g	Whole	No
	Monkey	Intravenous	100 μg/kg	Fetal (3)	6.6 ± 0.61^d^ pmol/g	1.0 pmol/g	Whole	No

Unless specified, mean total BPA ± SEM is reported. iv, intravenous; LLOQ, lower limit of quantification; ND, non detect; NM, not measured.

a Whole brain assumed as authors did not indicate region sampled.

b Aglycone BPA.

c Conjugated BPA.

d Maternal serum measurements; fetal serum not reported.

Most, if not all, studies have found some detectable amounts of BPA in the adult brain, but the blood: brain ratio is incredibly inconsistent when available for comparison. No study to date has performed cardiac perfusion to clear the brain of blood and its constituents, or attempted to correct for blood contamination. This suggests that there may be artificially inflated BPA estimates in the brain tissue. Authors often assume that the biologically active circulating BPA (aglycone) can easily and passively cross the adult BBB because of its lipophilic structure (Negri-Cesi, 2015; Santoro et al., 2019; Wang et al., 2019). Interestingly, the authors do not acknowledge that most substrates for the major BBB efflux proteins are lipophilic and contain some polar groups (Ek et al., 2012). One *in vivo* study showed that BPA can have weak modulatory effects on Breast Cancer Resistant Protein in rats (Nickel and Mahringer, 2014). Some *in vitro* evidence also suggests that BPA could be a P-glycoprotein substate (Mazur et al., 2012; Dankers et al., 2013); however, *in vivo* data are lacking. If BPA is interacting with efflux transporters like Breast Cancer Resistant Protein and P-glycoprotein, then several questions remain unanswered: is BPA crossing the BBB *because* of this interaction? Does this interaction allow for other endogenous or exogenous compounds to cross more easily? If so, could developmental exposure to BPA cause lasting increases in permeability of the BBB?

Unlike PFAS, the bisphenol literature does not often comment on the developing brain barriers. *In vivo* studies are limited, and brain concentrations often fall below the limit of detection (Domoradzki et al., 2003; Doerge et al., 2011; Patterson et al., 2013). Compared to other tissues, Doerge et al. (2011) found higher concentrations of aglycone BPA in the fetal rat brain following intravenous administration to dams; however, the level of aglycone BPA in fetal tissues dropped below the limit of detection if dams were dosed orally, indicating that the route of exposure can play an important role in tissue accumulation. The authors did not comment on how the chemical could traverse into the young brain. Uchida et al. (2002) estimated that BPA can reach the fetal brain within approximately 1 h for monkeys and 30 min for mice following subcutaneous injection to the mother. They concluded that the fetus is “indefensible” against BPA after it crosses the placenta, implying a lack of any additional barriers such as the BBB. In one developmental study, Mita et al. (2012) exposed mice to BPA from the first day of gestation through 1 week post birth and analyzed different tissues approximately 3 months after their last exposure. Despite having a short serum half-life in rodents (Doerge et al., 2011), authors reported brain tissue concentrations in the µg/g range (Mita et al., 2012). They also found that the male offspring of exposed mothers had the highest concentration of BPA in their brain tissue compared to other tissues, suggesting that BPA could have a longer half-life in the brain, or there was experimental contamination (Mita et al., 2012). Female offspring of exposed mothers did not exhibit the same trend. No serum BPA levels were reported, so conclusions are hard to draw (Mita et al., 2012). Much like the adult literature, the developmental neurotoxicology literature also contains many inconsistencies both within (Patterson et al., 2013) and across developmental studies.

Research regarding the degree to which bisphenols accumulate in the human fetus remains particularly uncertain (Corrales et al., 2015). Bisphenol analogues have been measured in cord blood (Liu et al., 2017; Kolatorova et al., 2018; Pan et al., 2020), with BPA having the highest concentration. BPA has been measured in human fetal cord blood at varying concentrations of 0.14–9.2 ng/ml (Corrales et al., 2015) and has been reported to reach concentrations five-fold higher in the amniotic fluid than in maternal serum in a Japanese cohort (Ikezuki et al., 2002). This is somewhat supported by *in vivo* pharmacokinetic work in sheep (Gingrich et al., 2019) in which BPA, but not other bisphenol congeners, were higher in fetal compartments. Concerningly, all tested bisphenols (BPA, BPS, and BPF) had a longer half-life in the fetus compared to the dam (Gingrich et al., 2019). However, other *in vivo* pharmacokinetic studies show amniotic fluid concentrations consistently lower than the corresponding maternal serum levels (Doerge et al., 2011; Patterson et al., 2013).

There is no general agreement on how much the embryo and fetus are truly exposed to bisphenols. Human fetal tissue characterization is especially challenging due to difficulties acquiring samples for analysis. As such, there are no publications to date that examine fetal brain concentrations. Both biomonitoring and *in vivo* studies have evidence to suggest that bisphenols can reach the fetus via maternal-placental transfer (Nishikawa et al., 2010; Gingrich et al., 2019). Thus, further study for the biodistribution and effects of bisphenol exposure in human neonates is needed. The developing brain is considered to be particularly sensitive to BPA (Pelch et al., 2017; Patisaul, 2020), and epidemiological studies suggest that neonatal exposure to BPA may be associated with altered neurodevelopment (see Ejaredar et al., 2017 and Mustieles and Fernandez 2020 for review). Additionally, current evidence suggests that circulating maternal BPA levels, a proxy for potential prenatal exposure, is more consistently associated with children’s neurobehavior than postnatal exposure (Mustieles and Fernandez, 2020). A large data gap exists investigating whether these changes in neurobehavior are associated with perturbations in the brain barriers, including BPA’s potential interaction with efflux proteins.

## Closing remarks

While it is true that the young brain is vulnerable to environmental contaminants (Diamanti-Kandarakis et al., 2009; Landrigan and Goldman, 2011; Axelrad et al., 2013; Gore et al., 2014; Landrigan et al., 2019; O'Shaughnessy et al., 2021), this vulnerability does not imply a lack of protection. The brain barriers are a fundamental defense against foreign compounds and necessary for homeostasis at all life stages, including development. The widespread belief that the barriers are absent or leaky in the embryo and fetus is not founded on cumulative evidence. Instead, this dogma was likely perpetuated following the publication of several studies with contested and now overturned conclusions, stating that the developing brain does not possess functional barrier systems. This has inadvertently led to a large data gap regarding how environmental contaminants may, or may not, penetrate the young brain.

The two chemical classes reviewed here, PFAS and bisphenols, are some of the most extensively studied pollutants in environmental toxicology. While epidemiological and experimental evidence suggests some of these chemicals may enter the brain, this is inconclusive. The bulk of data for PFAS shows that their concentrations in the central nervous system are lower compared to other tissues at both early and late life stages. This suggests that the brain barriers are active even in the fetus, and these chemicals are not freely diffusing from the blood and to the brain. Data for BPA are more variable, with a wide range of findings regarding brain concentration versus other tissues (including blood). Unfortunately, nearly all the toxicology studies reviewed possess a common flaw: blood contamination in the brain tissue. Without removal of the blood or accurately estimating its contamination, chemical measures in the brain parenchyma will be artificially inflated and data interpretation difficult. One could potentially estimate blood contamination with paired serum/plasma and brain tissue concentrations, but this would not be straightforward. Experimental methods to remove or address blood as a confounder exist (Saunders et al., 2015), but may be difficult for some toxicology laboratories to implement. The most attainable approach is likely transcardiac perfusion using a physiological buffer like saline, which would exsanguinate the brain before performing chemical measurements. Perfusion is a methodology commonly used for *in vivo* research (see Gage et al., 2012). However, the success of a perfusion is highly dependent on an individual’s skill, as the perfusate flow rate can be either too low or high, leading to inefficient blood removal and/or ruptured microvasculature. It is critical that experienced personnel perform the procedure and care is taken to mimic a physiologically relevant flow rate. Methods like autoradiography permit accurate quantification of compound transfer across the barriers and can show spatial distribution, but these require radioactivity and may pose safety concerns (Bickel, 2005). In all, methodologies more commonplace in neuropharmacology can lead us to experimental approaches that will permit more accurate chemical quantification in brain tissue. This will lead to more soundly supported conclusions regarding a chemical’s ability to enter the brain.

It should be noted that a xenobiotic crossing into the brain does not immediately classify it as neurotoxic. A chemical is only neurotoxic if it exhibits either direct or indirect effects on the central nervous system. As such, a chemical can also possess neurotoxic activity without entering the brain, like through mediation of the endocrine system (e.g., thyroid disruptors) and/or action on the peripheral nervous system (e.g., organophosphates and inhibition of acetylcholinesterase). Nevertheless, chemicals that cross into the brain with high efficacy should be prioritized for thorough *in vivo* testing, as this is likely an uncommon occurrence (Pardridge, 2016). This knowledge also represents an opportunity for innovation - new approach methodologies that estimate a chemical’s ability to bypass the brain barriers could represent new ways to rapidly prioritize compounds for their neurotoxicity risk. In addition to a chemical crossing into the brain and exerting toxicity, xenobiotics may also influence the barriers’ physical and metabolic properties. Interestingly, it has been hypothesized in the last decade that BBB disruption may act as the initiating trigger of many neurological disorders, as opposed to being a consequence of disease (Stanimirovic and Friedman, 2012). While there are very few environmental toxicology studies that address this possibility, this idea can be captured under the Developmental Origins of Health and Adult Disease (DoHAD) framework (Heindel et al., 2015), and is an important area of future research. In conclusion, the brain barriers are an often overlooked consideration in developmental neurotoxicology. We cannot assume that a compound can freely enter the developing brain in either humans or laboratory animals given the plethora of basic science data that shows otherwise. In the future, planning experiments to account for brain barrier activity will lead to more accurate estimates of chemical transfer into the brain and could impact hazard estimates.
